# COVID-19 and access to subsidized dental care according to household income. A register-based population study from Norway

**DOI:** 10.2340/aos.v84.44938

**Published:** 2025-12-15

**Authors:** Nan Jiang, Jonas Minet Kinge, Irene Skau, Jostein Grytten

**Affiliations:** a Department of Community Dentistry, University of Oslo, Blindern, Oslo, Norway; b Department of Health Management and Health Economics, University of Oslo and Norwegian Institute of Public Health, Oslo, Norway; c Department of Obstetrics and Gynecology, Institute of Clinical Medicine, Akershus University Hospital, Lørenskog, Norway

**Keywords:** Inequality, COVID-19, lockdown, dental care, household income

## Abstract

**Objective:**

The objective of this study was to examine inequalities in access to subsidized dental care according to household income before, during and after the COVID-19 lockdown in Norway.

**Material and methods:**

Our data encompassed all individuals aged 20 years and above (*n* = 3,977,747). The data were analyzed using a linear probability model. The outcome variable was whether an individual had received subsidized dental care, and the main independent variable was net household income. Analyses were carried out with and without the following control variables: gender, age (fixed effects), level of education, immigrant background and place of residence (fixed effects for county). Inequalities were measured using concentration indices.

**Results:**

The proportion of adults who received subsidized dental care was 3.10% before lockdown, 0.47% during lockdown and 2.15% after lockdown. The concentration indices were small. The unstandardized concentration indices (without control variables included) were: 0.04 before, 0.002 during and 0.04 after lockdown. The standardized concentration indices (with control variables included) were: 0.02 before, −0.007 during and 0.02 after lockdown. We carried out several supplementary analyses. They all supported our main findings.

**Conclusion:**

The lockdown did not lead to inequalities in access to subsidized dental care in Norway according to income.

## Introduction

On March 11 2020, the World Health Organization (WHO) announced COVID-19 as a pandemic [1]. By the end of the summer 2020, COVID-19 had spread to more than 200 countries, with nearly 25 million infected people and about 800,000 deaths [2]. In Norway, during March 2020, COVID-19 caused 46 deaths [3]. On March 12, the Norwegian Government introduced comprehensive policies to control the infection [4, 5]. Universities, schools, kindergartens and many other public facilities were closed down. Whenever possible, people had to work from home and avoid using public transport. People had to isolate themselves, travelling was restricted and populated areas were to be avoided. In public places, individuals had to keep a distance of at least 2 meters. During the spring and summer of 2020, the restrictions were gradually removed. From April 20^th^, schools, kindergartens and health institutions reopened. Recommendations about social distancing remained until the autumn (for a more detailed timeline of the different types of measures see Ursin et al. [6].

We examined the impact of the lockdown on inequalities in access to subsidized dental care for adults in Norway. Subsidized care covers about 20% of the total cost of dental treatment [7–9]. An important policy goal is to secure equal access to everyone who needs treatment [10–12]. Was this policy goal fulfilled during the lockdown and during the months after the restrictions were removed?

Within dentistry, so far most studies of the COVID-19 pandemic have focused on the clinical part of the infection, its transmission and the efficacy of different measures in reducing the risk of infection [13–16]. Several studies, from different countries, have found fewer dental visits during the lockdown compared to before lockdown [17–33]. Fewer studies have data about the period after lockdown [20, 21, 23, 25, 32, 33]. However, these studies show a gradual rise in the number of dental visits after society reopened [20, 21, 23, 25, 32, 33].

To our knowledge, there are few studies in which the effect of the COVID-19 pandemic on inequalities in access to dental services has been examined. The studies show conflicting results, for example the studies from the United Kingdom (UK) and the United States of America (US) [17, 21, 30–32]. One study from Australia shows that inequalities in access increased [25]. A limitation of some of these studies is that they lack data about the period after lockdown, thus the long-term impact of the lockdown on inequalities was not examined [30, 31].

In our study, we used a large and unique set of population register data from Norway. (A comprehensive description of the data is given in Jiang et al. [34]). Our data cover the whole adult population, encompassing about 4 million people. We have reliable data on the key variables, such as household income. Our analyses were carried out over three periods: before lockdown, during lockdown and after lockdown. Thus, we were able to study changes in inequalities due to the COVID-19 pandemic.

## Materials and methods

Our data file encompassed all individuals aged 20 years and above (*n* = 3,977,747). The way this data file was constructed and the different types of health registers that it was composed of, are described in Supplementary Appendix 1.

The data were analyzed using a linear probability model. The model is specified in Supplementary Appendix 1. The outcome variable was whether an individual had received subsidized dental care, and the main independent variable was net household income. This income variable was composed of labor income, capital income and all transfers from the government. It was adjusted to account for household composition using the OECD-modified equivalence scale [35, 36]. Analyses were carried out with and without the following control variables: gender, age (fixed effects), level of education, immigrant background and place of residence (fixed effects for county).

Results were presented for the following periods in 2020: before lockdown – 2^nd^ February – 11^th^ March, during lockdown – 12^th^ March – 19^th^ April and after lockdown – 20^th^ April – 28^th^ May. The length of each period was the same: 39 days.

Inequalities were measured using concentration indices. These are often used to measure socioeconomic inequality in health and health care utilization [37, 38]. These indices are in the range −1 to 1. The closer the indices are to zero, the smaller the inequalities. The way these indices are derived from the regression estimates from the linear probability model are given in Jiang et al. [31, 39]. A detailed description is also given in Supplementary Appendix 1.

We present results for the unstandardized concentration index (control variables not included) and the partial concentration index (control variables included) [37, 38]. In some analyses, we also present the index described by Erreygers which measures absolute inequality [40]. This is particularly useful when the outcome is binary, as in our case. A more detailed description of the differences between the three indices is given in Supplementary Appendix 1.

### Supplementary analyses

#### Margins plots

In a supplementary analysis, we estimated inequalities in access to subsidized dental care using margins plots. These plots were constructed from a logit model in which we assigned each individual to a decile, ranked from 1 to 10, based on net equalized household income (Supplementary Appendix 1). If access to subsidized dental care is independent of income, the estimates for each of the deciles would be small. Then the concentration indices would also be small.

#### Same number of weekdays in the three periods

In our main analyses, the number of weekdays^1^ in the three periods was not the same, because the number of public holidays was different. In the supplementary analyses, we adjusted the length of the periods before and after lockdown to make the number of weekdays the same as in lockdown (24 days).

### Analyses for 2019

We ran analyses of the relationship between equalized household income and the probability of having received subsidized dental care for the period 2^nd^ February – 11^th^ March 2019, that is, the same number of days as in the period before the lockdown in 2020.

Ideally, we wanted the concentration indices to be the same in 2019 as in the period before the lockdown in 2020. If they were the same, it would be reasonable to assume that the estimates for the period before the lockdown in 2020 were unbiased. If they were not the same, this would indicate that the estimates for the period before the lockdown in 2020 were biased. This could be because the lockdown was expected. Then, utilization of dental services may have increased in the period just before the lockdown, and the increase could be associated with household income.

#### Different types of dental services

The effect of the lockdown on the probability of receiving subsidized dental care may vary according to diagnosis. This was examined using a logit model. We examined the effects of the lockdown on the probability of obtaining subsidized dental care for each of the following groups:

1. Periodontal disease
2. Diseases and abnormalities in the mouth and jaw, excluding caries
3. The other diagnostic groups for which treatment costs are reimbursed by the National Insurance Scheme, merged into one category [8]. The reference group was composed of individuals who did not receive subsidized dental care.

#### Inequalities in access in subgroups of the population

The effects of the lockdown may vary for different subgroups of the population. We examined this by carrying out separate analyses for non-western immigrants and for residents in rural areas. People in these subgroups may face more barriers to subsidized dental care than the rest of the population, leading to inequalities. We classified people living in a municipality with less than 5,000 inhabitants as people living in a rural area. In Norway, nearly 50% of municipalities have less than 5,000 inhabitants. Seven percent of the population live in these municipalities [41].

## Results

### Descriptive statistics

The proportion of adults who received subsidized dental care was 3.10% before lockdown, 0.47% during lockdown and 2.15% after lockdown (Table 1). The 95% confidence intervals (CIs) did not overlap.

**Table 1:** Percentage of individuals who received subsidized dental care according to time period (*n* = 3,977,747).

Before lockdown (2nd February – 11th March)	Lockdown (12th March – 19th April)	After lockdown (20th April – 28th May)
3.10	0.47	2.15
[3.08 − 3.12]	[0.47 − 0.48]	[2.14 − 2.16]

In Table 2, we show key characteristics of the study population for those who had received subsidized dental care and for those who had not. A higher proportion of women than men had received subsidized dental care. Most subsidized dental care was provided to the following groups: women, people with university/college education, Norwegians and elderly people. The mean net equalized household income was about the same for those who had received subsidized dental care and for those who had not.

**Table 2:** Characteristics of the study population. Percentages and mean values for 2020 (*n* = 3,977,747).

Variables		Subsidized dental care	No subsidized dental care
Categorical variables:			
Gender	Male	46.2	50.3
		[46.1 − 46.3]	[50.2 − 50.3]
Education	Compulsory school education	21.3	20.7
		[21.2 − 21.4]	[20.6 − 20.7]
	Upper secondary education	45.3	40.4
		[45.1 − 45.4]	[40.3 − 40.4]
	University/college education	32.3	36.3
		[32.2 − 32.4]	[36.3 − 36.4]
Immigrant background	Norwegian	80.9	76.2
		[80.8 − 81.0]	[76.1 − 76.2]
	Western	9.4	12.9
		[9.3 − 9.5]	12.8 − 12.9]
	Non-western	9.7	11.0
		[9.6 − 9.8]	[10.9 − 11.0]
Continuous variables:			
Age (in years)		55.4	49.5
		[55.3 − 55.5]	[49.4 − 49.5]
Net household income (Euro)		45,719	44,280
		[45,611 − 45,828]	[44,232 – 44,328]

Number of individuals		521,084	3,456,663

Nearly 75% of all patients who received subsidized dental care had periodontal disease or abnormalities in the mouth and jaw (Supplementary Appendix 2, Table S1).

### Concentration indices

The concentration indices were small (Table 3). This was the case, irrespective of which type of index was used. These results show that inequalities in access to subsidized dental care were small.

**Table 3:** Different types of concentration index according to time period (*n* = 3,977,747). Length of each period: 39 days.

Type of concentration index	Before lockdown (2nd February – 11th March)	Lockdown (12th March – 19th April)	After lockdown (20th April – 28th May)
Unstandardized	0.038	0.002	0.044
	[0.035 to 0.041]	[−0.006 to 0.010]	[0.041 to 0.048]
Partial (standardized)	0.0165	−0.0068	0.0167
	[0.0159 to 0.0171]	[−0.0070 to −0.0066]	[0.0162 to 0.0172]
Corrected (Erreygers)	0.002	−0.00013	0.0014
	[0.001 to 0.003]	[−0.0004 to 0.00011]	[0.0009 to 0.0019]

The unstandardized indices varied from 0.002 (lockdown) to 0.044 (after lockdown). The 95% CI for the lockdown period did not overlap with the intervals for the periods before and after lockdown.

The partial indices were significantly lower than the unstandardized indices. The partial indices varied from −0.006 (lockdown) to 0.016 (before and after lockdown). The 95% CI for the lockdown period did not overlap with those for the periods before and after lockdown. For all three periods, the measures of absolute inequality [40] were almost zero.

### Supplementary analyses

#### Margins plots

**Figure 1:**
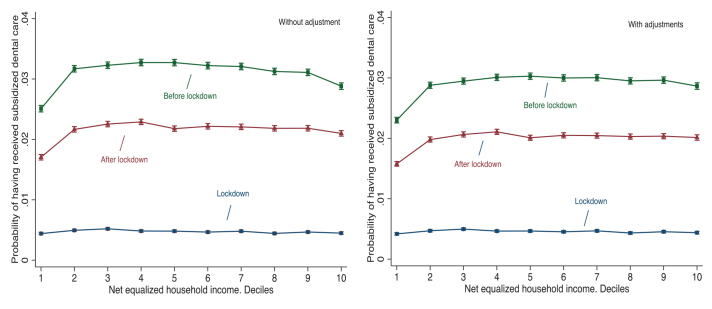
Probability of having received subsidized dental care according to time period and net equalized household income. Length of each period: 39 days (*n* = 3,977,747). Marginal probabilities. 95% Confidence Interval.

In Figure 1, we show margins plots estimated from the logit model (Supplementary Appendix 1, Equation (4)). The plots support our key findings shown in Table 3. The lines for most of the income deciles are nearly horizontal. The slope of the lines did not alter whether control variables were included or not included in the regression.

The lines were slightly steeper for the lowest income decile for the periods before and after lockdown. This indicates inequalities in access for people in this income group. However, the differences in the probability of obtaining subsidized dental care for individuals in the lowest and the highest deciles were small (less than 0.01 percentage points. See detailed results in Supplementary Appendix 2, Table S2). The CIs overlapped for most of the other deciles. In particular, this was the case in the estimations with control variables included (Supplementary Appendix 2, Table S3).

For all income deciles, the line for the lockdown period was well below the lines for the periods before and after lockdown. This shows that the probability of receiving subsidized dental care was lower in the lockdown period than in the other two periods. The line for the period before lockdown was above the line for the period after lockdown.

### Same number of weekdays in the three periods

In these analyses, the number of weekdays in each period was the same (Supplementary Appendix 2, Table S4). The estimates were only slightly lower than in the analyses in which the number of weekdays was not the same (Table 3).

### Analyses for 2019

During the period 2^nd^ February – 11^th^ March 2019, 1.5% of adults received subsidized dental care. The numbers for the concentration indices were (95% CIs in brackets):

- unstandardized: 0.0334 [0.0287 – 0.0382]
- partial: 0.0171 [0.0166 – 0.0175]
- corrected: 0.0010 [0.0006 – 0.0014]

These estimates were fairly similar to the estimates for the period in 2020 before the lockdown (Table 3).

### Different types of dental services

These analyses were carried out on samples in which the number of days in each period was the same. The overall pattern is that for all diagnostic groups, the concentration indices were small (Supplementary Appendix 2, Table S5). In particular, this is the case for the lockdown period. For all periods, the measures of absolute inequality [40] were close to zero.

#### Inequalities in access in subgroups of the population

There were no inequalities in access during the lockdown for non-western immigrants or for residents in rural areas. During the lockdown, for both subgroups the lines for the income deciles were horizontal (Supplementary Appendix 2, Figures S1 and S2). The CIs overlapped for all the income deciles (Supplementary Appendix 2, Tables S6–S7).

## Discussion

During the lockdown period, routine and non-urgent dental care was given low priority, including most of the treatment covered by the National Insurance Scheme. Dentists could still provide necessary and essential dental care, but then under strict regulations using protective equipment and following guidelines for infection control. The proportion of the adult population that received subsidized dental care was low (Table 1). This was a direct result of the regulations that were introduced to reduce the spread of infection. After lockdown, the proportion of the population that received subsidized dental care increased, but to a lower level than before lockdown. This finding is similar to several studies that have shown a gradual rise in the number of dental visits after society reopened [20, 21, 23, 25, 32, 33].

We examined inequalities in access to subsidized dental care using concentration indices (Supplementary Appendix 1). These indices are often used to examine inequalities in health care. Our findings show that it was possible to achieve the egalitarian aim of equality in service provision even during lockdown. This was a consistent finding both in our main analyses and in our supplementary analyses. Dentists treated their patients equally during lockdown. This implies that there was no need to introduce supply side measures aiming to equalize access for patients in different income groups.

Even before lockdown, inequalities in access to subsidized dental care were small (Table 3). Partly, this is because the supply of dentists has been adequate to meet demand for dental services. In Norway, the number of dentists in relation to the population is higher than in most countries in the world. The number of inhabitants per dentist is slightly above 1,000 [42–44]. Dentists are geographically evenly distributed [45]. The waiting time for a dental appointment is short [46, 47]. Thus, services have been easily available for those who needed subsidized dental care.

There are several countries in which the number of dentists per person-labor year is lower than in Norway. In these countries, there may have been inequalities in access to dental care before lockdown. Most likely, these inequalities in access would then also have been present during and after lockdown. In particular, this could be the case if patients had to pay for most of their dental treatment themselves, with no or little subsidized dental care. Thus, our results do not necessarily apply to countries in which inequalities in access existed before the lockdown.

The data from the Norwegian Health Economics Administration do not contain information about use of urgent dental services. Thus, we could not examine inequalities for urgent dental services according to income. This is a limitation of our study. During the lockdown, data on patients’ level of income and their use of urgent dental services were difficult to obtain. This may be one reason why there are no studies in which inequalities for urgent dental services according to income have been examined.

Our study has several strengths. First, the study was carried out using register data, in which the whole population of adults 20 years and over were included. In most of the previous studies, survey data or dental claims data have been used (for example see references 20, 21, 23, 25, 26, 31–33). These studies have their weaknesses. For example, they may suffer from different types of bias, such as sampling bias, non-response bias and recall bias [48–50]. Second, for each of the three time periods, we had reliable data about the use of the subsidy scheme for each respondent. Our data were detailed, so we could make the length of each period the same. Thus, we were able to study changes in inequalities as a result of the COVID-19 pandemic, using three time periods that were comparable. Third, in an additional analysis we included several control variables. Thus, it is unlikely that the results were biased due to unobservable characteristics that were correlated with the timing of the lockdown. Fourth, we carried out several supplementary analyses, such as estimating the concentration indices for different types of dental services. The results from these analyses supported our key results.

In conclusion, our study showed that the lockdown did not lead to inequalities in access to subsidized dental care. This result is important, as it provides insight into how the COVID-19 pandemic affected an important part of provision of dental services in Norway. Our results do not necessarily apply to other countries. In particular, this could be the case for countries in which inequalities in access existed before the lockdown.

## Supplementary Material

COVID-19 and access to subsidized dental care according to household income. A register-based population study from Norway

## Data Availability

The data for this project are owned by the Norwegian Directorate of Health and Statistics Norway. The authors are willing to give advice to researchers regarding procedures to apply for access to these data.

## Footnotes

1 Weekdays: total number of days in each period, minus weekends and public holidays.

## Statements and Declarations

Concept and design: JG and JMK. Acquisition, analyses and interpretation of data: JG, JMK and NJ. Data management: IS. Statistical analyses: NJ. Drafting of the manuscript: JG and NJ. Critical revision of the manuscript for important intellectual content: NJ, JG and JMK. Supervision: JG and JMK.
