# Supplementary material for: COVID-19 and access to subsidized dental care according to household income. A register-based population study from Norway
Source: Acta Odontol Scand. 2025 Dec 15;84:44938. doi: 10.2340/aos.v84.44938 (PMC12712853; doi:10.2340/aos.v84.44938)
Supplement: COVID-19 and access to subsidized dental care according to household income. A register-based population study from Norway [file AOS-84-44938-s1.pdf]

Supplementary material has been published as submitted. It has not been copyedited or typeset by Acta Odontologica Scandinavica.

Supplementary Appendix 1

Construction of the data file

The way the data file has been constructed is described by Grytten and Skau (2017, 2018, 2020) [1-3]. A short summary is as follows:

Reimbursement payments for dental care for people 20 years and above are administered by the Norwegian Health Economics Administration [4]. All adults who receive subsidized dental treatment are registered with this body. All persons who live in Norway have a unique personal identification number. This made it possible to merge the data from the Norwegian Health Economics Administration with the following data registers in Statistics Norway:

- Norwegian Registry for Personal Taxpayers: income for all people with a tax obligation in Norway [5]
- Norwegian Standard Classification of Education: highest education of all persons living in Norway [6]
- Population Register: place of residence (municipality) of all persons living in Norway [7]
- Population Register on Immigration: country of origin of first and second generation immigrants to Norway [8]

By merging data from the Norwegian Health Economics Administration with data from Statistics Norway, our final data file encompassed the whole adult population.

Regression model and indices

$$Subsidized\_Dental\ Care_i = \alpha + \beta y_i + \delta x_i + \epsilon_i \qquad (1)$$

where $Subsidized\_Dental\ Care_i$ is a binary dependent variable taking the value one if an individual i, had received subsidized dental care, and zero otherwise. Net household income is defined by y_i, and x_i is a row vector of control variables.

The unstandardized and the partial concentration indices:

We calculated the unstandardized concentration index and the partial concentration index using the following formula:

$$CI = \frac{\beta\mu^{y}}{\mu^{m}}C^{yy} + \frac{\delta\mu^{\chi}}{\mu^{m}}C^{xy} + \frac{\mu^{\varepsilon}}{\mu^{m}}C^{\varepsilon y} \quad (2)$$

where β and δ are the coefficients from Equation (1), μ^{y} is the mean of the income variable, μ^{χ} is the mean of the covariates, μ^{ε} is the mean of the error term, and μ^{m} is the proportion who had received subsidized dental care. C^{yy}, C^{xy} and $C^{\varepsilon y}$ are concentration indices for income (unstandardized), the covariates (partial) and the error term, respectively.

The corrected concentration index (Erreygers, 2009) [9]:

We calculated the corrected concentration index using the following formula:

$$CCI = \frac{4\mu^{m}}{a-b} \times CI = 4\mu^{m}\mathrm{CI} \quad (3)$$

In Equation (3), μ^{m} is the mean of the outcome variable, *a* and *b* are the maximum and minimum values of the outcome variable, which are 1 and 0 in our case. Hence, the corrected standardized CCI can be written as $4\mu^{m}\mathrm{CI}$.

Key differences between the three indices (see also Jiang et al. (2022, 2024) [10,11]):

The unstandardized concentration index takes into account both the direct effect that household income has on the probability of having received subsidized dental care, and the indirect effects that are transmitted through the intervening variables [12]. In our model, the indirect effects would be the component of the association between the probability of having received subsidized dental care and income that is explained by the control variables.

In the literature, it is common to estimate the partial concentration index [12, 13]. This is a measure of income-related inequality in health after removing the indirect effects of income that are transmitted through intervening variables. In our case, the partial concentration index would be a measure of the direct effect of income on the probability of having received subsidized dental care. We used the method of indirect standardization to estimate the partial concentration index [12].

The unstandardized concentration index and the partial concentration index measure relative inequality. An alternative approach would be to measure absolute inequality, which quantifies the absolute differences between income groups according to the probability of having received subsidized dental care. Wagstaff (2005) and Erreygers (2009) have developed indices that measure absolute inequality [9,14]. These indices are particularly useful when the outcome is binary, as in our case. With binary outcomes, the minimum and maximum values of the concentration indices depend on the mean of the outcome variable [15]. This complicates comparison of the values of the concentration indices across populations in which the mean of the outcome variable varies. In our study, the proportion of individuals who received subsidized dental care was lower during the lockdown period than before or after lockdown. Therefore, to take account of the fact that the mean of the outcome variable varied across the samples, we also measured inequalities using the corrected concentration index proposed by Erreygers (2009) [9].

Margins plots –logit model

We estimated the following logit model:

$$\frac{P(Subsidized\_Dental\ Care_i = 1)}{P(\ Subsidized\_Dental\ Care_i = 0)} = \varphi Net\ equalized\ household\ income_i + Control\ variables_i + \varepsilon_i \qquad (4)$$

References

1. Grytten J, Skau I. The impact of education on the probability of receiving periodontal treatment. Causal effects measured by using the introduction of a school reform in Norway. Soc Sci Med. 2017;188:128-136.

2. Grytten J, Skau I. Do patients with more education receive more subsidized dental care? Evidence from a natural experiment using the introduction of a school reform in Norway as an instrumental variable. Med Care 2018;56:877-882.

3. Grytten J, Skau I. Educational inequalities in access to fixed prosthodontic treatment in Norway. Causal effects using the introduction of a school reform as an instrumental variable. Soc Sci Med. 2020;260:113105.

4. Norwegian Health Economics Administration. About Helfo. [cited 2025 Aug 14]. Available from: https://www.helfo.no/om-helfo/om-helfo/about-helfo

5. Statistics Norway. Tax for personal tax payers. [cited 2025 Aug 14]. Available from: https://www.ssb.no/en/inntekt-og-forbruk/skatt-for-personer/statistikk/skatt-for-personer

6. Barrabés N, Østli GK. Norsk standard for utdanningsgruppering 2016. Revidert 2000. Dokumentasjon. Oppdatert 2016. Notater 2016/30. Oslo: Statistics Norway; 2016.

7. Statistics Norway. Immigrants and Norwegian-born to immigrant parents. [cited 2025 Aug 14]. Available from: https://www.ssb.no/en/befolkning/innvandrere/statistikk/innvandrere-og-norskfodte-med-innvandrerforeldre

8. Statistics Norway. Population and housing census. [cited 2025 Aug 14]. Available from: https://www.ssb.no/en/befolkning/artikler-og-publikasjoner/statistics-from-the-population-and-housing-census-2011-to-continue

9. Erreygers G. Correcting the concentration index. J Health Econ. 2009;28:504-515.

10. Jiang N, Grytten J, Kinge JM. Inequality in access to dental services in a market-based dental care system: A population study from Norway 1975-2018. Community Dent Oral Epidemiol. 2022;50:548-558.

11. Jiang N, Kinge JM, Skau I, Grytten J. Does subsidized orthodontic treatment reduce inequalities in access? Evidence from Norway based on population register data. Community Dent Oral Epidemiol. 2024;52:232-238.

12. Gravelle H. Measuring income related inequality in health: standardisation and the partial concentration index. Health Econ. 2003;12:803-819.

13. O'Donnell O, van Doorslaer E, Wagstaff A, Lindlow M. Analyzing health equity using household survey data: a guide to techniques and their implementation. Washington: The World Bank; 2007.

14. Wagstaff A. The bounds of the concentration index when the variable of interest is binary, with an application to immunization inequality. Health Econ. 2005;14:429-432.

Supplementary Appendix 2

Table S1

Distribution of individuals who received subsidized dental care according to diagnostic group in different time periods.

Length of each period: 39 days.

	Before lockdown (2nd February - 11th March)		Lockdown (12th March - 19th April)		After lockdown (20th April – 28th May)	
Diagnostic group	Number	Percent	Number	Percent	Number	Percent
Periodontal disease	66 625	54.0	5 340	28.4	42 858	50.2
Diseases and abnormalities in the mouth and jaw, excluding caries	23 519	19.0	7 642	40.7	17 550	20.5
All other groups	33 157	26.9	5 808	30.9	25 046	29.3
Number of individuals	123 301	100	18 790	100	85 454	100

Table S2

Probability of having received subsidized dental care according to net equivalized household income (in deciles) and time period. Without adjustments. Marginal probabilities. 95% CI in brackets. Length of each period: 39 days.

Net equivalized household income (deciles)	Before lockdown (2nd February - 11th March)	Lockdown (12th March - 19th April)	After lockdown (20th April – 28th May)
1	0.0251<br>[0.0246 – 0.0256]	0.0044<br>[0.0042 − 0.0046]	0.017<br>[0.0166 − 0.0175]
2	0.0317<br>[0.0312 – 0.0322]	0.0049<br>[0.0047 − 0.0052]	0.0217<br>[0.0212 − 0.0221]
3	0.0323<br>[0.0317 – 0.0328]	0.0052<br>[0.0050 − 0.0054]	0.0225<br>[0.0221 − 0.0230]
4	0.0327<br>[0.0322 – 0.0333]	0.0048<br>[0.0046 − 0.0050]	0.023<br>[0.0225 − 0.0234]
5	0.0327<br>[0.0322 – 0.0333]	0.0048<br>[0.0046 − 0.0050]	0.0218<br>[0.0213 − 0.0222]
6	0.0322<br>[0.0317 – 0.0328]	0.0047<br>[0.0044 − 0.0049]	0.0222<br>[0.0217 − 0.0226]
7	0.0321<br>[0.0315 – 0.0326]	0.0048<br>[0.0046 − 0.0050]	0.0221<br>[0.0216 − 0.225]
8	0.0313<br>[0.0307 – 0.0318]	0.0044<br>[0.0042 − 0.0046]	0.0218<br>[0.0214 − 0.0223]
9	0.0311<br>[0.0306 – 0.0316]	0.0047<br>[0.0044 − 0.0049]	0.0218<br>[0.0214 − 0.0223]
10	0.0288<br>[0.0283 – 0.0294]	0.0045<br>[0.0043 − 0.0047]	0.021<br>[0.0206 − 0.0214]

Table S3

Probability of having received subsidized dental care according to net equivalized household income (in deciles) and time period. With adjustments. Marginal probabilities. 95% CI in brackets.
Length of each period: 39 days.

Net equivalized household income (deciles)	Before lockdown (2nd February - 11th March)	Lockdown (12th March - 19th April)	After lockdown (20th April – 28th May)
1	0.023<br>[0.0226 − 0.0235]	0.0042<br>[0.0040 − 0.0044]	0.0158<br>[0.0154 − 0.0162]
2	0.0288<br>[0.0283 − 0.0293]	0.0047<br>[0.0045 − 0.0049]	0.0198<br>[0.0194 − 0.0202]
3	0.0295<br>[0.0290 − 0.0300]	0.005<br>[0.0048 − 0.0052]	0.0206<br>[0.0202 − 0.0211]
4	0.0301<br>[0.0296 − 0.0306]	0.0046<br>[0.0044 − 0.0049]	0.0211<br>[0.0206 − 0.0215]
5	0.0303<br>[0.0298 − 0.0308]	0.0047<br>[0.0044 − 0.0049]	0.0201<br>[0.0197 − 0.0205]
6	0.03<br>[0.0295 − 0.0305]	0.0045<br>[0.0043 − 0.0047]	0.0205<br>[0.0201 − 0.0209]
7	0.03<br>[0.0295 − 0.0306]	0.0047<br>[0.0045 − 0.0048]	0.0205<br>[0.0200 − 0.209]
8	0.0295<br>[0.0290 − 0.0300]	0.0043<br>[0.0041 − 0.0045]	0.0203<br>[0.0200 − 0.0209]
9	0.0296<br>[0.0291 − 0.0302]	0.0045<br>[0.0043 − 0.0048]	0.0204<br>[0.0199 − 0.0208]
10	0.0287<br>[0.0281 − 0.0292]	0.0044<br>[0.0042 − 0.0046]	0.0201<br>[0.0197 − 0.0206]

Table S4
Different types of concentration index according to time period.
Length of each period: 24 weekdays.

Type of concentration index	Before lockdown	Lockdown	After lockdown
Unstandardized	0.036<br>[0.033 − 0.040]	0.002<br>[-0.006 − 0.010]	0.044<br>[0.040 − 0.049]
Partial (standardized)	0.0151<br>[0.0145 − 0.0156]	-0.0068<br>[-0.0070 − -0.0066]	0.0170<br>[0.0165 − 0.0174]
Corrected (Erreygers)	0.0016<br>[0.0011 − 0.0022]	-0.00013<br>[-0.0004 − 0.00011]	0.0012<br>[0.0008 − 0.0017]

Table S5
Different types of concentration index according to diagnostic group and time period.
The length of each period: 39 days.

Diagnostic group	Before lockdown (2nd February - 11th March)	Lockdown (12th March - 19th April)	After lockdown (20th April – 28th May)
Periodontal disease			
Unstandardized	0.065 [0.061 − 0.070]	0.04 [0.02 − 0.05]	0.083 [0.078 − 0.089]
Partial (standardized)	0.0268 [0.0264 − 0.0272]	0.0119 [0.0118 − 0.0120]	0.0324 [0.0320 − 0.0328]
Corrected (Erreygers)	0.0018 [0.0014 − 0.0023]	0.00006 [-0.00006 − 0.0002]	0.0014 [0.0011 − 0.0018]
Diseases and abnormalitiesin the mouth and jaw, excluding caries			
Unstandardized	0.031 [0.023 − 0.038]	0.0001 [-0.0128 − 0.0132]	0.032 [0.024 − 0.041]
Partial (standardized)	0.0324 [0.0321 − 0.0326]	0.0054 [0.0053 − 0.0056]	0.0301 [0.0299 − 0.0304]
Corrected (Erreygers)	0.0008 [0.0005 − 0.0011]	0.00004 [-0.00011 − 0.00019]	0.0005 [0.0003 − 0.0008]
All other groups			
Unstandardized	-0,0109 [-0.0171 − -0.0046]	-0.0288 [-0.0437 − -0.0138]	-0.0119 [-0.0191 − -0.0047]
Partial (standardized)	-0,0146 [-0.0149 − -0.0143]	-0.0401 [-0.0402 − -0.0400]	-0.0190 [-0.0192 − -0.0187]
Corrected (Erreygers)	-0.0005 [-0.0008 − -0.0002]	-0.0002 [-0.0004 − -0.0001]	-0.0005 [-0.0008 − -0.0002]

Table S6

Probability of having received subsidized dental care according to net equivalized household income (in deciles) and time period. Non-western immigrants. With adjustments. Marginal probabilities. 95% CI in brackets. Length of each period: 39 days.

Net equivalized household income (deciles)	Before lockdown (2nd February - 11th March)	Lockdown (12th March - 19th April)	After lockdown (20th April – 28th May)
1	0.0203 [0.0194 – 0.0211]	0.0037 [0.0033 − 0.0041]	0.0126 [0.0119 − 0.0133]
2	0.0255 [0.0243 – 0.0267]	0.0047 [0.0041 − 0.0052]	0.0166 [0.0157 - 0.0176]
3	0.0281 [0.0267 – 0.0295]	0.0049 [0.0043 − 0.0055]	0.0181 [0.0170 - 0.0193]
4	0.0290 [0.0274 – 0.0306]	0.0050 [0.0043 − 0.0056]	0.0199 [0.0186 − 0.0212]
5	0.0288 [0.0271 – 0.0306]	0.0050 [0.0042 − 0.0057]	0.0183 [0.0169 − 0.0197]
6	0.0289 [0.0270 - 0.0308]	0.0042 [0.0034 - 0.0049]	0.0196 [0.0180 − 0.0212]
7	0.0289 [0.0269 – 0.0310]	0.0046 [0.0037 − 0.0054]	0.0193 [0.0176 - 0.0210]
8	0.0293 [0.0271 – 0.0316]	0.0051 [0.0042 − 0.0061]	0.0195 [0.0177 − 0.0213]
9	0.0269 [0.0246 – 0.0291]	0.0047 [0.0037 − 0.0056]	0.0178 [0.0160− 0.0196]
10	0.0266 [0.0242 – 0.0291]	0.0041 [0.0032 − 0.0051]	0.0195 [0.0174 − 0.0216]

Table S7

Probability of having received subsidized dental care according to net equivalized household income (in deciles) and time period. Rural residents. With adjustments. Marginal probabilities. 95% CI in brackets. Length of each period: 39 days.

Net equivalized household income (deciles)	Before lockdown (2nd February - 11th March)	Lockdown (12th March - 19th April)	After lockdown (20th April – 28th May)
1	0.0203<br>[0.0189 – 0.0217]	0.0034<br>[0.0029 − 0.0040]	0.0128<br>[0.0117 − 0.0139]
2	0.0241<br>[0.0227 - 0.0255}	0.0034<br>[0.0029 - 0.0040]	0.0162<br>[0.0151 − 0.0174]
3	0.0264<br>[0.0250 – 0.0279]	0.0040<br>[0.0034 − 0.0045]	0.0166<br>[0.0154 − 0.0177]
4	0.0275<br>[0.0259 – 0.0290]	0.0033<br>[0.0028 − 0.0039]	0.0181<br>[0.0169 − 0.0193]
5	0.0278<br>[0.0263 – 0.0294]	0.0034<br>[0.0028 − 0.0039]	0.0169<br>[0.0157 − 0.0182]
6	0.0273<br>[0.0257 - 0.0288]	0.0038<br>[0.0032 − 0.0044]	0.0175<br>[0.0163 − 0.0189]
7	0.0273<br>[0.0257 – 0.0289]	0.0042<br>[0.0035 − 0.0048]	0.0176<br>[0.0163 - 0.0188]
8	0.0268<br>[0.0252 – 0.0284]	0.0031<br>[0.0026 − 0.0037]	0.0191<br>[0.0177 − 0.0204]
9	0.0283<br>[0.0265 – 0.0301]	0.0035<br>[0.0029 − 0.0041]	0.0172<br>[0.0158 − 0.0185]
10	0.0258<br>[0.0239 – 0.0278]	0.0032<br>[0.0025 − 0.0039]	0.0167<br>[0.0151 − 0.0183]

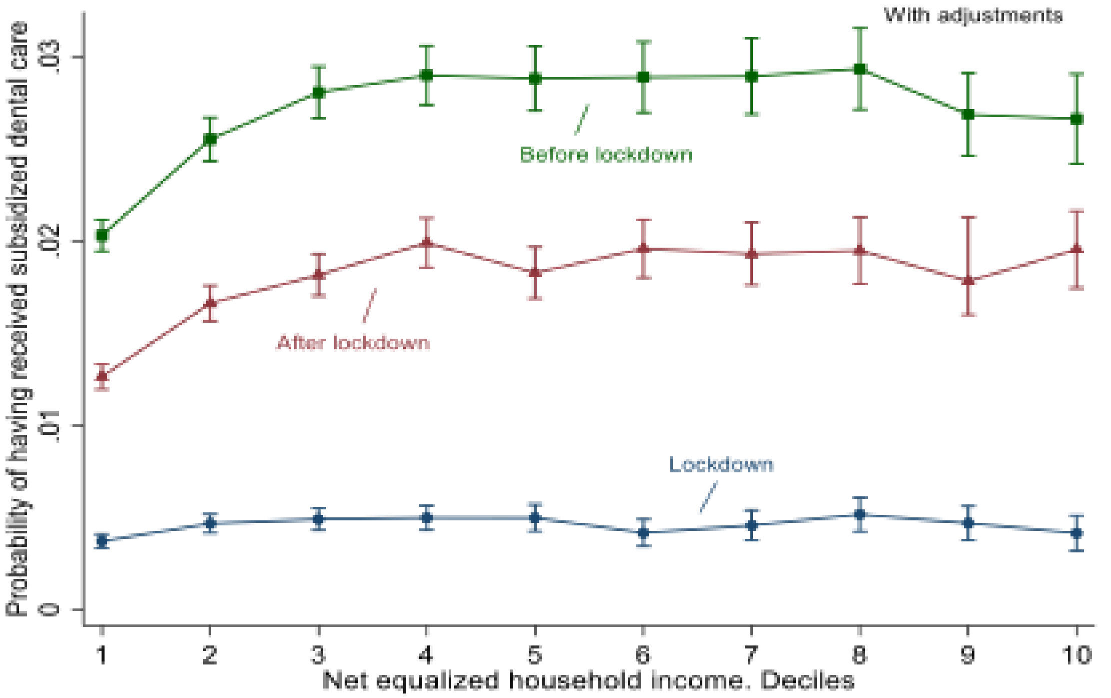

Figure S1. Probability of having received subsidized dental care according to time period and net equalized household income.

Non-western immigrants.Length of each period: 39 days (n=429 699). Marginal probabilities. 95% CI.

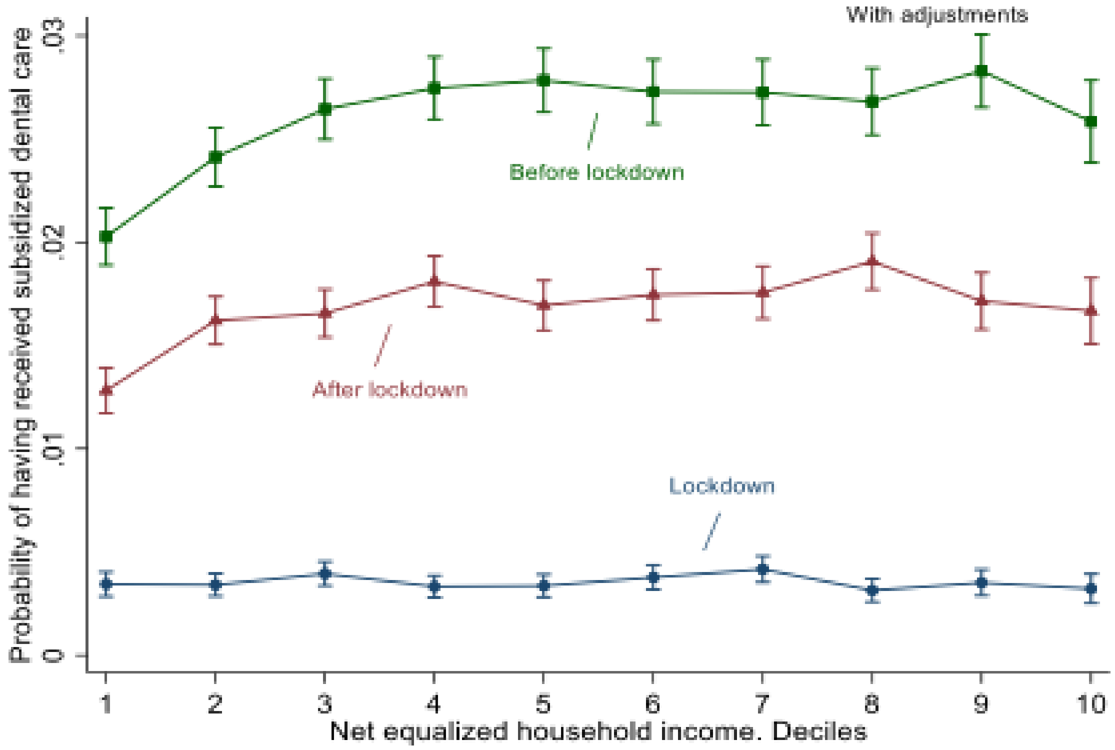

Figure S2. Probability of having received subsidized dental care according to time period and net equalized household income.

Rural residents. Length of each period: 39 days (n=387 175). Marginal probabilities. 95% CI.
